# Cellulose Nanofibrils-Reinforced Pectin Membranes for the Adsorption of Cationic Dyes from a Model Solution

**DOI:** 10.3390/polym16060724

**Published:** 2024-03-07

**Authors:** Alenka Ojstršek, Selestina Gorgieva

**Affiliations:** Institute of Engineering Materials and Design, Faculty of Mechanical Engineering, University of Maribor, Smetanova 17, 2000 Maribor, Slovenia; selestina.gorgieva@um.si

**Keywords:** cellulose nanofibrils, pectin, membrane, cationic dyes, adsorption, dye removal

## Abstract

In the presented research, a facile, one-step method for the fabrication of cellulose nanofibrils/pectin (CNFs/PC) membranes is described, which were tested further for their ability to remove cationic dyes from the prepared model solutions. For this purpose, ten membranes were prepared with different quantities of CNFs and PC with/without citric acid (CA) or CaCl_2_ as mediated crosslinking agents, and they were characterised comprehensively in terms of their physical, chemical, and hydrophilic properties. All the prepared CNFs/PC membranes were hydrophilic with a Water Contact Angle (WCA) from 51.23° (without crosslinker) up to 78.30° (CaCl_2_) and swelling of up to 485% (without crosslinker), up to 437% (CaCl_2_) and up to 270% (CA). The stability of membranes was decreased with the increase in PC; thus, only four membranes (M1, M2, M3 and M5) were stable enough in water after 24 h, and these were additionally applied in the adsorption trials, using two structurally different cationic dyes, i.e., C.I. Basic Yellow 28 (BY28) and C.I. Basic Blue 22 (BB22), in four concentrations. The highest total surface charge of M3 (2.83 mmol/g) as compared to the other membranes influenced the maximal removal efficiency of both dyes, up to 37% (BY28) and up to 71% (BB22), depending on the initial dye concentration. The final characteristics of the membranes and, consequently, the dye’s absorption ability could be tuned easily by changing the ratio between the CNFs and PC, as well as the type and amount of crosslinker.

## 1. Introduction

Coloured wastewaters from textile dyeing and printing plants are composed of numerous toxic, mutagenic, carcinogenic and non-degradable compounds of different origins and properties, including a mixture of diverse dyestuffs, pigments, organic matter, heavy metals, auxiliaries, chemicals, etc., and, thus, present severe environmental pollution if they are not treated properly before discharge into watercourses [1]. Moreover, coloured effluents could reduce the water transparency, leading to a decrease in the light penetration, photosynthesis and gas solubility of the water, which influences both water organisms and human health negatively [2]. Cationic (basic) dyestuffs are a class of synthetic dyes used mainly for the dyeing of acrylic fibres at acidic pH. The acrylic fibres market size reached around 1.6 million tonnes in the year 2022 [3], and the market is projected to register a Compound Annual Growth Rate (CAGR) of 3.7% during the 2023–2032 period [4]. Based on the increasing demand for acrylic fibre in the wool apparel segment (blended textiles), together with a rising trend in e-commerce for furniture and upholstery, it is expected that the quantity of coloured wastewaters from acrylic dye houses will increase, as well as the environmental issues accordingly.

As stated in [5,6], adsorption is the most popular technique for the removal of non-degradable organic compounds (e.g., dyes) and heavy metals in low concentrations from diverse and complex effluents, owing to its simplicity, ease of operation and low cost. The efficiency rate of the adsorption process is affected by numerous parameters, i.e., the type of adsorbent (physical, morphological, topological, and chemical features), the conditions during the adsorption process (absorbent dosage, pH, temperature, contact time, the initial concentration of pollutant and the presence of other contaminants) and the nature of the pollutant [7,8].

Many studies in recent years have investigated the potential of cellulose-based composites, fibres, membranes and aerogels as dye adsorbents, since cellulose is the most abundant biomass resource in the world. It is easy to obtain, ecologically friendly, renewable, biodegradable, low cost, etc. [9]. In particular, nanoscale cellulosic materials, such as cellulose nanocrystals (CNCs) and cellulose nanofibrils (CNFs), show superior affinity towards residual dyestuffs in wastewater treatment systems, due to their high length-to-diameter ratio, high specific surface area, and the abundance of chemical groups on their surface (hydroxyl and carbonyl), which help to adsorb positively charged pollutants [10].

CNFs are produced through the mechanical disintegration/defibrillation of cellulose pulp [11], and, therefore, consist of a bundle of micrometres-long entangled fibrils with nano-sized diameters in the range of 5 up to 100 nm, forming a gel-like structure in aqueous suspensions [12,13]. Moreover, they have low weight, low thermal expansion, high stiffness and excellent strength (1.6–3 GPa) and modulus (100–160 GPa). Thus, CNFs have been used widely to prepare high-strength, high-performance composite materials, including membranes for wastewater decolouration [12,14].

Pak et al. [13] fabricated all-carbohydrate-based membranes via the in situ hydrothermal carbonisation of glucose in the presence of CNF for the removal of Methylene Blue (MB), Orange G (OG) and Vitamin B12. The as-prepared membranes showed an excellent rejection rate just for cationic dye MB (92.77%). The rejection rates of neutral VB12 and anionic OG were as low as 1.12 and 6.19%, respectively, due to their poor electrostatic interactions with the membrane. Wu et al. [14] constructed an acid-resistant chitosan/CNF composite membrane via a gel-casting method for the adsorption of MB. The results proved that it had excellent acid-resistant properties and 14.71 mg/g of adsorption capacity for MB at pH 1.22, as well as excellent recyclability (about 85% of desorption rate) after five adsorption–desorption recycles. Gorgieva et al. [15] and Maleš et al. [16] revealed the highly effective performance of water-resistant biobased CMC/CNF membranes as adsorbents for the removal of different cationic dyes, i.e., MB and C.I. Basic Blue 47 and C.I. Basic Yellow 29, respectively. Other recently published studies on nanocellulose-based adsorptive membranes for the treatment of coloured wastewaters have been gathered in [7,17].

Another biobased, anionic, soluble, non-starch polysaccharide that has acquired great attention due to its diverse applications is pectin, due to its nontoxicity, biodegradability and renewability [18]. It can be extracted easily from the primary cell walls of the peel and pulp of numerous fruits, such as citrus peel, apple pomace, sugar beets, mango, etc. [19]. Since pectin contains a lot of electron-rich functional groups, i.e., carboxyl, hydroxyl, and acylamino groups, which can form an electrostatic interaction with an organic cation, it can be employed effectively for the removal of positively charged dyes from coloured wastewaters [8]. To overcome its limited thermal stability and mechanical properties in composites, pectin is usually mixed with different polymers and/or nanoparticles in the form of aerogels, films, hydrogels, nanocomposites, beads, membranes, etc. [19].

Al-Gorair et al. [19] successfully formed a pectin/acrylic acid/CNC nanocomposite by γ-irradiation for the remediation of Methylene Blue (MB) dye from a model solution. They concluded that the adsorption capacity increases with the increase in MB concentration up to 70 mg/L at pH 9, irrespective of the temperature used. Nesic et al. [18] reported composite films based on amidated pectin (AP) with a montmorillonite (MMT) content of 10, 30 and 50% for the adsorption of Basic Yellow 28. They found out that the adsorption capacity increases with the increase in MMT content, i.e., from 85 mg/g (AP/MMT 10%) up to 103 mg/g (AP/MMT 30%) and up to 126 mg/g (AP/MMT 50%), when the initial concentration of dye was set to 30 mg/L.

To the best of our knowledge, there is no such study reporting the application of a CNF/PC membrane as an adsorbent for the removal of commercially essential basic dyes used for the dyeing of acrylic textiles. Therefore, the aim of this research was to fabricate stable polysaccharide-based membranes using different ratios of CNFs and PC with/without a crosslinker (CA or CaCl_2_) and characterise them in terms of their physical, chemical and hydrophilic features. Subsequently, the adsorption behaviour of membranes was evaluated systematically against two structurally different cationic dyestuffs, C.I. Basic Yellow 28 and C.I. Basic Blue 22, in four concentrations. These two dyes were selected due to their commercial availability, disclosed chemical structure, different chemical structure (azo and anthraquinone), different shade and saturation.

Since as-prepared CNF/PC membranes possess many attractive features, such as sustainability, biocompatibility, low cost, compressibility and ease of fabrication, they were revealed to have potential for application in textile wastewater treatment systems.

## 2. Experimental Section

### 2.1. Materials

**Figure 1:**
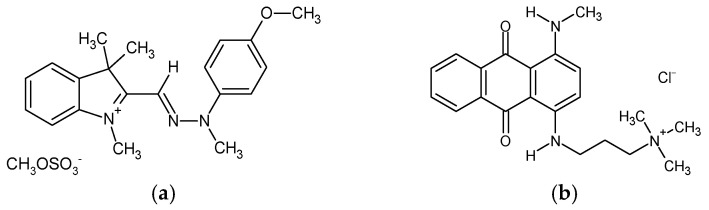
The chemical structure of the applied dyes: (**a**) C.I. Basic Yellow 28; and (**b**) C.I. Basic Blue 22.

Cellulose nanofibrils (CNFs) in the form of 3.0 wt.% aqueous gel were kindly supplied by the University of Maine (Orono, ME, USA). According to the supplier’s information, the CNFs’ specific surface area reached 31–33 m^2^/g (BET analysis), with a nominal fibre diameter of 20–50 nm and a density of 1.0 g/cm^3^. The pectin (PC) was purchased from Caesar & Loretz GmbH (Hilden, Germany), and the anhydrous citric acid (CA, ≥99.5%, M_w_ = 192.12 g/mol), calcium chloride (CaCl_2_, ≥96.0%, M_w_ = 110.98 g/mol), sodium hydroxide (NaOH, 97%, M_w_ = 40 g/mol) and hydrochloric acid (HCl, 37%, M_w_ = 36.46 g/mol) from Sigma Aldrich (St. Louis, MO, USA). All the chemicals were used as received, without further purification. Deionised (DI) water was applied in the preparation of all trials. Two structurally different cationic dyes were employed for the batch adsorption experiment, namely, methine dye C.I. Basic Yellow 28 (BY28, M_w_ = 433.5 g/mol) and anthraquinone dye C.I. Basic Blue 22 (BB22, M_w_ = 352.4 g/mol) (Figure 1), both purchased from the American Association of Textile Chemists and Colorists (New York, NY, USA).

### 2.2. Preparation of the Membranes

Firstly, 2% *w*/*v* of PC was dispersed in DI water by means of a magnetic stirrer under moderate stirring conditions (200 rpm), for 60 min, at an ambient temperature. After that, 1.5% *w*/*v* of CNFs was homogenised in a separate glass baker using a high-speed stirrer, Eurostar 20 (IKA GmbH, Staufen, Germany), for 60 min at 600 rpm, in order to avoid the presence of large, aggregated CNF bundles. Shortly before mixing the CNF and PC components, two water solutions of 5% *w*/*v* CA and 10% *w*/*v* CaCl_2_ were prepared separately. Finally, the as-prepared dispersions/solutions were mixed in the pre-defined amount (as shown in Table 1) and poured into glass Petri dishes which were 90 mm in diameter, and then, they were dried at 60 °C for 24 h. The dry membranes were washed 3 times with DI water to remove any remaining unfixed compounds and dried again at room temperature for 24 h to gain a constant weight. In addition, all membranes were comprehensively characterised as described in Section 2.4.1.

### 2.3. Batch Adsorption Experiment

The shake-flask experiments were conducted with the aim of evaluating the adsorption ability of an individual CNF/PC membrane for the removal of two structurally different cationic dyes in four concentrations. Coloured solutions were prepared by the dilution of an individual cationic dye, BY28 or BB22, into DI water in concentrations of 5, 15, 25 and 50 mg/L. The prepared membranes were cut into four parts and weighed. Each part was put into a 250 mL Erlenmeyer flask, together with 100 mL of an individual solution, where the pH was adjusted to pH 4 using 0.1 M NaOH or 0.1 M HCl. All the flasks were sealed and agitated on an orbital shaker, Promax 2020 (Heidolph Instruments GmbH & Co. KG, Schwabach, Germany), at 100 ± 2 rpm for 24 h. All the trials were accomplished in triplicate at an ambient temperature of 22 ± 2 °C. Afterwards, the membranes were taken out from the flasks and weighed. In addition, the adsorption was measured in the remaining solutions, and the percentage of colour removal and the amount of adsorbed dyestuff were calculated concerning its initial concentration in the solution.

### 2.4. Analytical Procedure

#### 2.4.1. Characterisation of the Membranes

Attenuated Total Reflectance–Fourier Transform Infrared (ATR–FTIR) spectroscopic measurements of membranes were made to determine the functional groups responsible for the dyestuff uptake, using an FT-IR/NIR/FIR Spectrophotometer Spectrum 3 (Perkin Elmer, Waltham, MA, USA) with a Golden Gate ATR attachment. The transmittance spectra were obtained within the range of 4000−550 cm^−1^, with the air spectrum subtraction performed in parallel as a background, employing 16 scans and a resolution of 4 cm^−1^.

The pH-potentiometric titration of diversely prepared CNF/PC membranes was carried out, in order to evaluate their surface charge, by employing a dual-burette automatic titrator, T70 (Mettler Toledo, Zurich, Switzerland), filled with 0.1 M HCl and 0.1 M KOH titrants. The titration was accomplished between pH 2.5 and 11 in a forward (acidic to alkaline) and backward (alkaline to acidic) manner, using an inert (N_2_) atmosphere. A detailed description of the procedure is given elsewhere [16].

The sessile drop method was employed to evaluate the hydrophilic/hydrophobic character of the prepared membranes. Herein, an individual membrane was set on a horizontal table on a Goniometer (DataPhysics Instruments GmbH, Filderstadt, Germany). A micro-drop with a volume of 0.3 µL of MilliQ water was poured onto the sample surface. A clear image of the drop was transferred directly through a CCD camera showing the drop profile. The Water Contact Angle (WCA) was determined from the tangent to the drop at the three-phase contact. All the WCA measurements were performed in duplicate on the top and bottom sides of the membrane, from which the mean value was calculated.

With the aim of determining the ability of water uptake, all the membranes were cut into 1 cm^2^ pieces, and afterwards, they were immersed into 30 mL of DI water. The pieces were taken out of the water at different time intervals (15, 30, 45 or 60 min), and then, the excess surface water was wiped off immediately by filter paper, and the membranes’ pieces were weighed. The percentage of swelling was calculated according to Equation (1) as follows:

(1) $$\mathrm{Swelling}\;(\%)=\frac{W_{w(t)\;\;}-W_{d}}{W_{d}},$$

where *W_w(t)_* is the weight of the wet membrane at time (*t*); and *W_d_* is the weight of the dry membrane.

A portable pH meter, MA 235 (Mettler Toledo, Zurich, Switzerland), together with the Standard [20] was employed to measure the pH values in the waters during the water uptake experiment, as well as in the coloured model solutions at the beginning of the adsorption trial.

#### 2.4.2. Analysis of the Coloured Model Solutions

The model solutions that included cationic dye were analysed during the batch adsorption experiment by measuring the absorbance at a wavelength of an individual dye’s absorption maximum, i.e., for BY28 at 439 nm and for BB22 at 588 nm, using a UV/Vis spectrophotometer, Cary 60 (Varian, Columbus, OH, USA), according to the Standard [21], employing a special measuring probe with 10 mm optical length. In addition, the absorbances of two solutions, BY28 and BB22, in an initial concentration of 25 mg/L, were monitored online over time (5, 10, 20, 30, 60, 120 and 180 min). The concentration of the remaining dye in an individual solution after the completion of an adsorption experiment was calculated using the Beer–Lambert law (Equation (2)), the percentage of dye’ removal (*R*) from the coloured solution according to Equation (3), and the amount of adsorbed dye (*Q*) per weight of individual membrane (mg/g) according to Equation (4) as follows:

(2) A=ε·C·l

(3) $$R=\frac{C_{0}-C}{C_{0}}\cdot 100\%$$

(4) $$Q=\frac{{(C}_{0}-C)\cdot V}{W}$$

where *A* is the absorbance; *ε* is the molar extinction coefficient obtained from the calibration curve for the individual dye at a wavelength of maximal absorption (L/g·cm); *l* is the optical pathway (mm); *C*_0_ is the initial concentration of the dye in the coloured solution (mg/L); *C* is the concentration of the dye after the adsorption trial (mg/L); *V* is the initial volume of the coloured solution (L); and *W* is the weight of the used membrane (g).

## 3. Results and Discussion

### 3.1. Characterisation of Membranes

**Figure 2:**
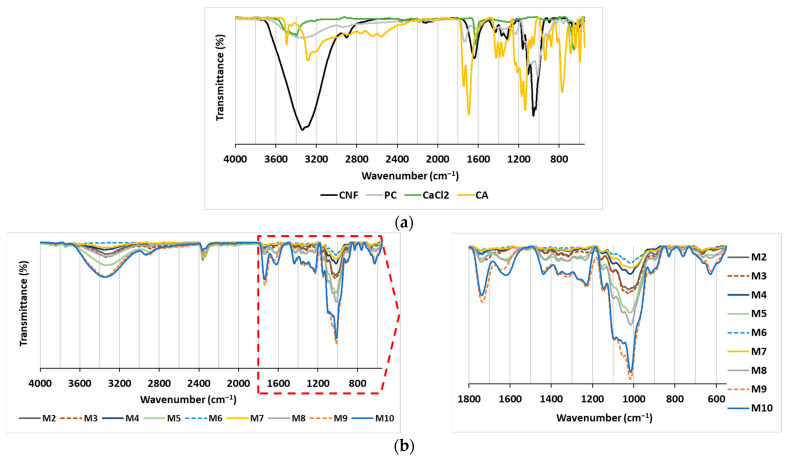
ATR-FTIR spectra of (**a**) the main components (CNF, PC, CA and CaCl_2_); and (**b**) the membranes (M2–M10).

The objective of the present study was to assess the effectiveness of polysaccharide-based membranes as adsorbents for the removal of two structurally different cationic dyes from model solutions. The rationality behind the variations made in membrane preparation was to identify the effect of different additions of CNF and PC as nanofibrous and charge-rich polymeric components, respectively, and CA or CaCl_2_ as covalent or ionic-bond-forming crosslinkers on membrane stability and adsorption capacity and to potentially identify the most suitable composition to deliver the highest adsorption efficiency. For this purpose, nine CNF/PC membranes (M2–M10) were prepared by changing the ratio between the content of CNF and PC and by adding a crosslinker (CA or CaCl_2_) or not, as proposed in Table 1. The first membrane (M1) was composed entirely of CNF, i.e., 32 g of 1.5% *w*/*v* of CNFs diluted in DI water, whereas the PC control (without CNF and crosslinkers) was excluded from experimental design, as PC does not possess film-forming capacity on its own. Both CNF and PC compounds were selected because of their easy accessibility and low cost. Several analytical techniques were used for the analysis of the membranes in order to provide qualitative or quantitative information about their chemical composition, total charge, hydrophilic features and swelling that could influence the membranes’ stability in water treatment systems, as well as their adsorption capability for dyes. The ATR-FTIR transmittance spectra of CNF/PC membranes were recorded within the region of 4000–550 cm^−1^ and are gathered in Figure 2.

Figure 2a depicts an FTIR spectral pattern with characteristic peak positions for the CNF, PC, CA and CaCl_2_ compounds used in the preparation of the CNF/PC membranes. The CNF-associated spectral line depicts the OH-related vibrations at 3500–3200 cm^−1^, C–H asymmetrical stretching in CH_2_ at ~2900 cm^−1^, glycosidic bonds in the region of 1100–930 cm^−1^ and a “fingerprint” region attributed to vibrations of the C–O–C bridges [19,22]. Due to its carbohydrate nature, the PC contains the same peaks as described for CNF, with the addition of C=O stretching at 1736 cm^−1^, ionic carboxyl group (COO−) stretching at 1613 cm^−1^, carboxylate group symmetric stretching at 1439 cm^−1^, side-chain vibrations at 1229 cm^−1^, and C–O stretching at 1014 cm^−1^ in PC [19,22]. The typical CA-related bands are OH-related bands in 3500–3200 cm^−1^, COO− stretching at 1638 cm^−1^, C-OH stretching at 1107.5 cm^−1^ and CH_2_ rocking at 662.7 cm^−1^ [16,23]. Typical bands related to CaCl_2_ are positioned in 3500–3400 cm^−1^ region, assigned to asymmetric O–H stretching, 2164 cm^−1^ for symmetric O–H stretching, and H–O–H bending vibration bands at 1629 and 1618 cm^−1^ due water to water presence [24].

By combining the CNF and PC in the membranes (Figure 2b—M1, M4 and M7), no new transmittance peak appeared, mainly revealing the physical crosslinking and electrostatic interaction of both compounds, as explained in [25]. When CA as a crosslinker was incorporated into CNF/PC membranes (M2, M5, M8), a broad peak at 1750 cm^−1^ was observed (the peak at ∼1615 cm^−1^ diminished), confirming the CA-mediated CNF coupling with PC by the formation of ester bonds between the carboxyl groups of CA and the hydroxyl groups of CNFs and PC. The esterification reaction was accelerated by a low pH or Lewis acids [15,26]. Moreover, some peaks were shifted or diminished in the region of 1430–1200 cm^−1^ (depending on the CNF/PC ratio), which is attributed to the carboxylate groups’ symmetric stretching. The band with strong intensity at ∼1014 cm^−1^, mainly associated with the C–O stretching vibrations of PC, was diminished in M7, M8 and M9, and, instead, the peak at ∼1028 cm^−1^, typical for CNF, became apparent, due to the lowest quantity of PC and the highest dosage of CNF as compared to all the other membranes. In addition, a broad peak was noticed for C–Cl vibrations at 667 cm^−1^ in M3, M6 and M9, which partly overlapped with the rocking C–H vibrations of CNF and PC [19]. Moreover, the vibration peak at 1735 cm^−1^ was reduced significantly in comparison to the pure CNF/PC membranes (M1, M4 and M7), indicating the interaction between Ca from the CaCl_2_ and carboxyl groups of the PC.

**Figure 3:**
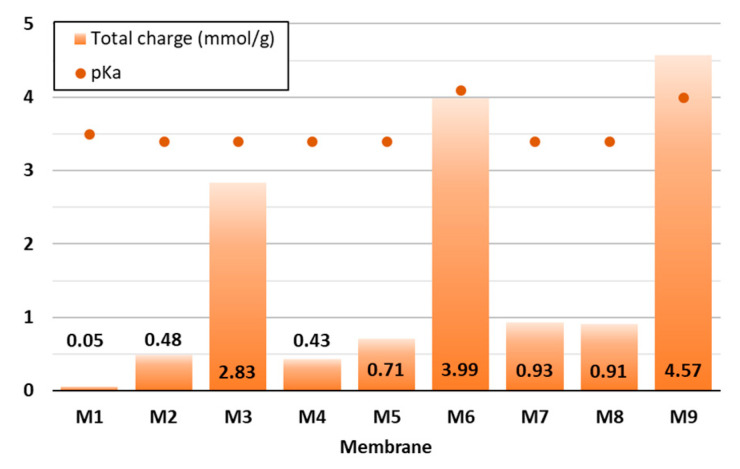
Potentiometric data of the CNF and CNF/PC membranes—total surface charge and pKa.

Since the surface charge of membranes is considered as a key factor affecting their adsorption ability, it was determined in the present study by means of potentiometric titration (Figure 3), which was based on the quantification of the ionised functional groups present in the organic material [27]. Diversely prepared CNF and CNF/PC membranes were titrated at a pH between 2.5 and 11.

As can be seen from Figure 3, 32 g of 1.5% *w*/*v* of CNFs (M1) had the lowest total surface charge (0.05 mmol/g), which is consistent with the results gained by [15]. The higher the PC content was, the higher the negative charge was, i.e., up to 1.27 mmol/g in M1 (24 g PC/8 g CNF). This agrees with the results reported by [28]. Remarkably, the addition of CA influenced the enlargement of the total charge by up to 3.3 mmol/g or 360% (M9), 3.06 mmol/g or 429% (M6) and 2.39 mmol/g or 658% (M3), as compared to the membranes without the crosslinker, which were M4, M7 and M10, respectively. The increase in total charge per mass was due to the ionisation of the carboxylic functional groups present in the structure of the mentioned mixtures, as also confirmed by the FTIR analysis of the membranes. The pKa of the disintegrated CNF/PC membranes was between 3.3 and 4.1—positively charged at lower pHs and negatively charged at higher pHs. Consequently, the pH of the wastewater during adsorption could affect the degree of ionisation of the CNF/PC membranes and their interaction with the cationic dyes.

**Figure 4:**
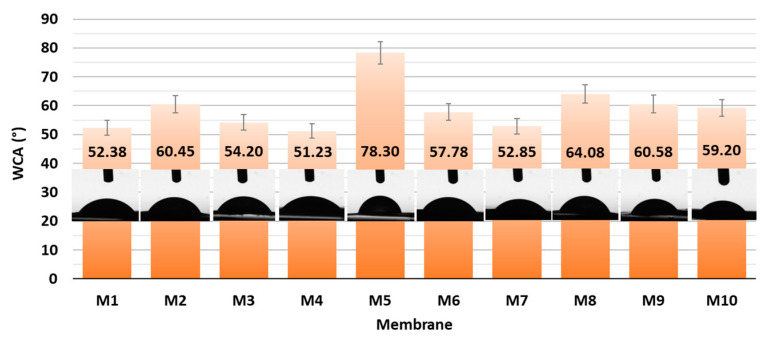
WCA of CNF and CNF/PC membranes with corresponding photographs.

With the aim of elucidating the role of different amounts of CNF and PC, as well as the type of crosslinker on the hydrophilic features, all the as-prepared membranes were evaluated via Water Contact Angle (WCA) measurement using goniometry. The gained results are presented graphically in Figure 4.

**Figure 5:**
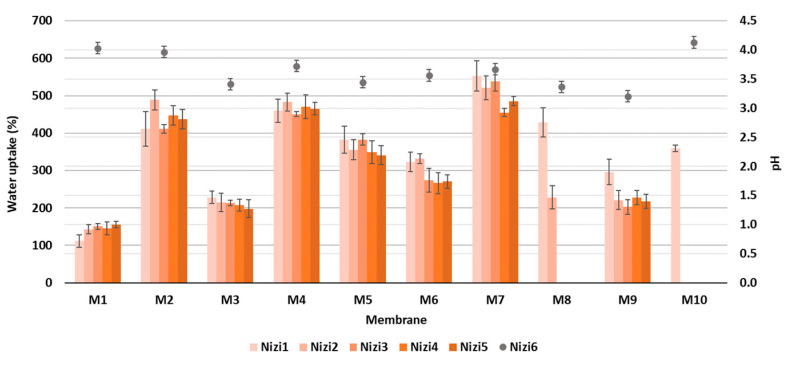
The percentage of water uptake at different time intervals (primary y-axis) and pH after 360 min (secondary y-axis).

From the results of the goniometric measurements in Figure 4, it can be concluded that all the prepared CNF/PC membranes were hydrophilic, since their WCAs were lower than 90°. This can be explained by the fact that both CNFs and PC are hydrophilic in nature, with a high number of hydroxyl functional groups in their structures, which is also confirmed by the FTIR results in Figure 2a. In general, the lowest WCAs were measured on the membranes without crosslinkers, i.e., 59.20 ± 2.96°, 52.85 ± 2.64° and 51.23 ± 2.56° for M10, M7 and M4, respectively. The most deviating was M5 with the lowest wettability (WCA 78.30 ± 3.92), revealing the most successful crosslinking with fewer remaining free hydroxyl groups. Besides functional groups, the contact angle of an individual membrane was affected significantly by its surface morphology and roughness [16]. Accordingly, the differences between the WCAs of membranes with different CNF/PC ratios, as well as between different crosslinkers, were minor. The hydrophilic character of the CNF/PC membranes also influences the water uptake, and, further, the adsorption capacitance; hence, the swelling behaviour of the diversely composted membranes in DI water was evaluated at different time intervals at a pH between 3.3 and 4.2, as shown in Figure 5.

As can be perceived from Figure 5, the percentage of water uptake increased for all the prepared membranes after over 360 min of immersion in DI water, due to the abundant carboxyl or hydroxyl functional groups. The enormous uptake in the first 15 min of the process (up to 653%) was stabilised after 45 min, when the equilibrium was reached. Two membranes, M8 and M10, were not stable enough, and disintegrated after 15 and 30 min, respectively; therefore, the obtained results were not relevant, and these membranes were not used further in the adsorption trial. In general, the highest swelling after 6 h was noticed for the CNF/PC membranes without crosslinkers (M4 and M7) of up to 485%, which is in accordance with the WCA results, followed by CNF/PCs crosslinked with CaCl_2_ (M2 and M5) of up to 437% and CNF/PCs crosslinked with CA (M3, M6, M9) of up to 270%, depending on the ratio between the CNF and PC. Similar results with maximum water uptake in the first minute (up to 400%) were reported by [29] for different types and thicknesses of CNF membranes. For the removal of dyes from water, it is very important that membranes have both maximum swelling, as well as high stability during the whole adsorption trial. In membranes M4 and M7 (without crosslinkers) with high swelling capacity, the mechanical stability of the network was reduced, forming fractures in the membranes’ structures and consequently resulting in their disintegration after 24 h. The addition of a crosslinker formed a denser network, as explained in [30], which was harder to access by water, lowering the percentage of swelling accordingly. Moreover, the protonation of anionic CA at low pH accelerated the hydrogen bonding formation and, thus, restricted the swelling capability of the membrane [19]. In addition, the stability of the membranes rose, and the swelling decreased by increasing the CNF/PC ratio, since the CNF narrowed the voids in the nanocomposite network and rendered the structure denser [19]. For the comparison with CNF/PC membranes, the pure CNF membrane (M1), consisting of 32 g of 1.5% *w*/*v* of CNFs, had the lowest water uptake, i.e., 155% after 6 h of immersion in DI water.

### 3.2. Adsorption of Cationic Dyes

**Figure 6:**
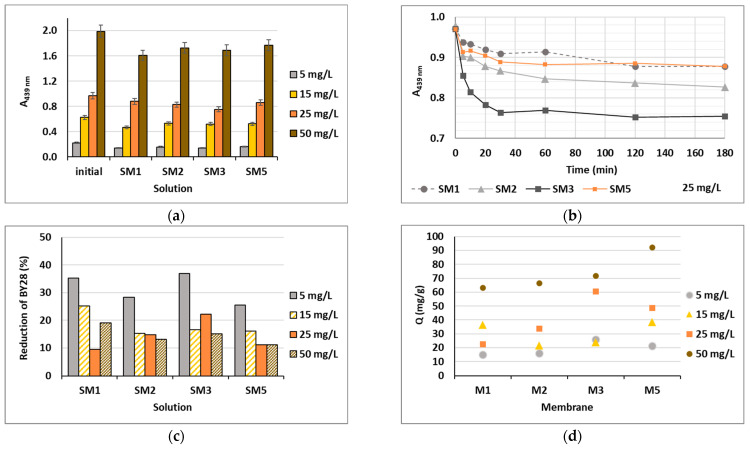
Cationic dye BY28: (**a**) absorbance (A) in the initial model solutions (initial) and in the solutions after 24 h of a batch experiment at λ_max_ = 439 nm; (**b**) on-line absorbance during the batch experiments at a dye concentration of 25 mg/L; (**c**) percentage of dye reduction after 24 h; and (**d**) the amount of adsorbed dye on membrane (Q).

**Figure 7:**
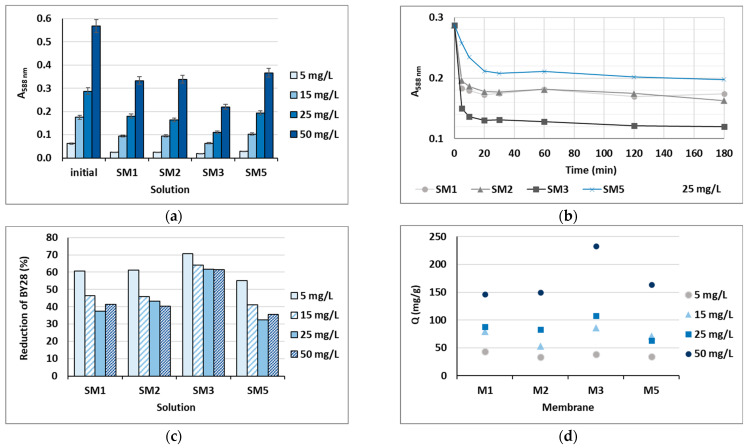
Cationic dye BB22: (**a**) absorbance (A) in the initial model solutions (initial) and in the solutions after 24 h of a batch experiment at λ_max_ = 588 nm; (**b**) percentage of reduction after 24 h; (**c**) on-line absorbance during a batch experiment at a dye concentration of 25 mg/L; and (**d**) the amount of adsorbed dye on the membrane (Q).

**Figure 8:**
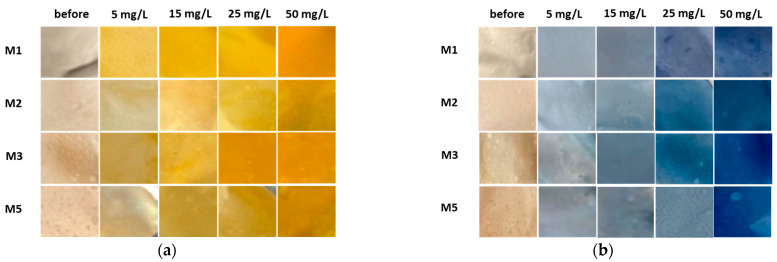
Corresponding smartphone photographs of membranes before and after the adsorption trial employing (**a**) BY28 and (**b**) BB22.

Based on the water uptake results, barely four membranes of the ten (M1, M2, M3 and M5) were stable enough in water after 24 h, and these were additionally employed in the shake-flask adsorption trials as adsorbents. Four model solutions were prepared using two cationic dyes in four initial concentrations, which were 5, 15, 25 and 50 mg/L. The results gained are presented in Figure 6 for BY28 and in Figure 7 for BB22, and the corresponding photographs of membranes after the adsorption trial for both dyes is shown in Figure 8. The abbreviations SM1, SM2, SM3 and SM5 mean solutions after the shake-flask adsorption trials employing the individual membranes M1, M2, M3 or M5.

As can be seen from Figure 6a and Figure 7a, BY28 is a more saturated dye with approximately 3.5–times higher absorbance in an initial model solution (initial) as compared to BB22 at the same concentration, and, thus, noticeably coloured wastewaters, even at an exceedingly low concentration. In addition, the adsorption of BY28 and BB22 was successful to different extents on different membranes, as can be perceived visually from Figure 8a,b, respectively. The maximal removal efficiency of both dyes was attained using M3, i.e., up to 37% (BY28—Figure 6b) and up to 71% (BB22—Figure 7b), depending on the initial concentrations of model solutions. M3 had the highest total surface charge, i.e., 2.83 mmol/g, in comparison to the other inspected membranes (M1, M2 and M5), affecting its superior adsorption ability, as explained in Section 3.1 under Figure 3.

As noticed from Figure 6c and Figure 7c, the higher the initial concentration of the dye was, the lower the percentage of dye removal from the model solution was, regardless of the membrane composition and the dye’s chemical structure. On the contrary, the membranes’ adsorption abilities were increased when the initial concentration increased, although this relation was not linear, as is evident from Figure 6d and Figure 7d. The calculated amount of adsorbed dye per weight of M3 was from 25.8 up to 71.8 mg/g (BY28), and from 37.8 up to 232.6 mg/g (BB22). This increase in adsorbed dye onto the membrane by enhancing dye concentration can be attributed to the active interaction between the membrane and dye, due to the accelerated diffusion of dye molecules onto the adsorbent [18].

**Figure 9:**
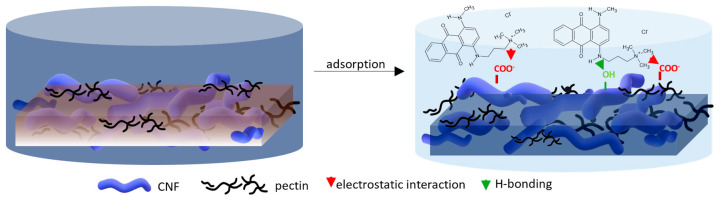
Proposed adsorption mechanism of cation dye BB22 removal by a CNF/PC membrane.

For a better understanding of the dye’s adsorption mechanism for an individual membrane over time, the absorbance at 439 nm (BY28) or 588 nm (BB22) was measured online for up to 3 h using a dye solution of 25 mg/L, and the obtained results are shown in Figure 6b and Figure 7b, respectively. The highest drop in absorbance (the highest colour reduction) was observed in the first 20 min of the experiment, which is in accordance with the results obtained by [10]. After that, the change in absorbance was negligible, irrespective of the employed membrane or dye. As explained by Li et al. [8] and Pak et al. [13], the adsorption process of positively charged dyestuffs by the negatively charged pectin- or cellulose-based adsorbents above the pKa value is mainly based on their strong electrostatic interactions (physisorption), as shown schematically in Figure 9. In addition, hydrogen bonds can be formed between N atoms (hydrogen bond receptors) in dye molecules and hydroxyl groups in pectin/cellulose molecules, and also, new conjugates can be established between C=C-bonds in dye molecules and hydroxyl and carboxyl groups in pectin/cellulose molecules (chemisorption) [8]. The better removal efficiency of BB22 in comparison to BY28 dye can also be attributed to its lower molar mass, facilitating more adsorbate movement through the membrane pores, and, accordingly, leaving the adsorption sites available for binding, as explained in [31].

## 4. Conclusions

In the presented study, ten fully polysaccharide-based membranes were fabricated successfully in a one-step method using different ratios of CNFs and PC, without or with a crosslinker (CA or CaCl2), and the most stable were further used as adsorbents for the removal of two structurally different cationic dyes from a model solution. All membranes show a hydrophilic character, with WCA = 51.23° up to 78.30°, and superior water uptake (270% up to 485%). The best adsorption capability for both dyes was attained by employing membrane 3 (M3), which had the highest total surface charge as compared to other stable membranes. The removal efficiency was up to 37% (BY28) and up to 71% (BB22), depending on the initial dye concentration. Therefore, the results show that CNFs influence the overall stability of the membrane, PC assists the cationic dye adsorption and CA increases the stability of CNF/PC membranes as well as remarkably enlarges the total surface charge. In general, the developed CNF/PC membranes revealed their potential for upscaling them for application in textile wastewater treatment systems, since they are sustainable, biocompatible, have low costs and are easy to manufacture. Further work will focus on the optimisation of the adsorption parameters, as well as on the removal of some other charged pollutants, e.g., metal ions.

## Figures and Tables

**Table 1:** The weight (g) of (PC and CNF) dispersions and volume (mL) of (CA and CaCl_2_) solutions, premixed for preparation of respective membranes.

Membrane	CNF [g]	PC [g]	CA [g]	CaCl_2_ [g]
1	32			
2	24	8		4
3	24	8	4	
4	24	8		
5	16	16		4
6	16	16	4	
7	16	16		
8	8	24		4
9	8	24	4	
10	8	24		

## Data Availability

Data are contained within the article.
